# Labor and social protection gaps impacting health of non-standard workers: An international study

**DOI:** 10.1093/eurpub/ckac129.437

**Published:** 2022-10-25

**Authors:** S Kvart, I Cuervo, V Gunn, S Baron, T Bodin, PWR Consortium

**Affiliations:** 1 Unit of Occupational Medicine, Institute of Environmental Medicine, Karolinska Institutet, Stockholm, Sweden; 2 Commoner Center Health and Environment, Queens College, City University of New York, Flushing, USA; 3 Lawrence S. Bloomberg Faculty of Nursing, University of Toronto, Toronto, Canada; 4 MAP Centre for Urban Health Solutions, Li Ka Shing Knowledge Institute, Toronto, Canada; 5 Center for Occupational and Environmental Medicine, Stockholm Region, Stockholm, Sweden

## Abstract

**Background:**

Labor regulations and social protection structures are intended to protect workers, but the needs of those in precarious and non-standard employment (NSE) are often missed, which may negatively impact health and well-being. The aim of this research is to document how workers in NSE in six countries - Belgium, Canada, Chile, Spain, Sweden, and the US - with varying policy contexts experience aspects of employment that are linked to health.

**Methods:**

We employed a mixed methods approach for this study. To understand policy contexts, we analyzed country-level labor regulatory and social protection frameworks using 2019 Organization for Economic Cooperation and Development data. To understand the experiences of workers in NSE, we conducted 250 in-depth interviews with workers at different levels of employment precariousness between January and June 2021.

**Results:**

Overall, European countries have the most social expenditures and North American countries have the weakest labor market regulations. In all these varying contexts, workers in NSE reported multiple unmet needs, e.g., inadequate paid sick and parental leave and unemployment compensation. These unmet needs occur due to various barriers, including poor enforcement, legal loopholes, or required minimum employment time. Workers’ living accommodations are also affected, as home financing or rental contracts are dependent on permanent employment. In response, they tended to rely disproportionately on individual or family resources for financial and social support rather than on government or employer resources.

**Conclusions:**

Findings suggested that diverse labor regulatory and welfare regime contexts are unsupportive of workers in NSE due to multiple gaps in policies essential to public health. The shifting of responsibility for key employment and social supports to individuals and their families is likely to increase health inequities for workers in NSE.

**Key messages:**

• Our study documents multiple policy gaps affecting key employment-related social determinants of health among workers in NSE.

• This occurred across diverse labor and social structure contexts in six countries.

